# “Worldwide Network for Blood & Marrow Transplantation (WBMT) special article, challenges facing emerging alternate donor registries”

**DOI:** 10.1038/s41409-019-0476-6

**Published:** 2019-02-18

**Authors:** Mahmoud Aljurf, Daniel Weisdorf, Feras Alfraih, Jeff Szer, Carlheinz Müller, Dennis Confer, Shahrukh Hashmi, Nicolaus Kröger, Bronwen E. Shaw, Hildegard Greinix, Mohamed A. Kharfan-Dabaja, Lydia Foeken, Adriana Seber, Syed Ahmed, Areej El-Jawahri, Moheeb Al-Awwami, Yoshiko Atsuta, Marcelo Pasquini, Amr Hanbali, Hazzaa Alzahrani, Shinichiro Okamoto, Eliane Gluckman, Mohamad Mohty, Yoshihisa Kodera, Mary Horowitz, Dietger Niederwieser, Riad El Fakih

**Affiliations:** 1 Adult Hematology/HSCT, Oncology Centre, King Faisal Specialist Hospital & Research Centre, Riyadh, Saudi Arabia; 2 University of Minnesota, Minneapolis, MN USA; 3 Clinical Haematology at Peter MacCallum Cancer Centre and Royal Melbourne Hospital, VIC, 3050 Australia; 4 Zentrales Knochenmarkspender-Register ZKRD, Ulm, BW Germany; 5 Center for International Blood and Marrow Transplant Research, National Marrow Donor Program/Be The Match, Minneapolis, USA; 6 University Hospital Hamburg, Hamburg, Germany; 7 Center for International Blood and Marrow Transplant Research (CIBMTR), Medical College of Wisconsin, Milwaukee, WI USA; 8 Medical University of Graz, Division of Hematology, Graz, Austria; 9 Mayo Clinic, Jacksonville, FL USA; 10 World Marrow Donor Association, Leiden, Netherlands; 11 Hospital Samaritano de São Paulo, Sao Paulo, Brazil; 12 Massachusetts General Hospital, Boston, MA USA; 13 Japanese Data Center for Hematopoietic Cell Transplantation, Nagoya, Aichi Japan; 14 Asia-Pacific Blood and Marrow Transplantation Group (APBMT), Tokyo, Japan; 15 Eurocord Hôpital Saint-Louis and University Paris Diderot, Paris, France; 16 Monacord Centre scientifique de Monaco, Monaco, France; 17 Hopital Saint-Antoine, Sorbonne University, INSERM UMRs 839, Paris, France; 18 Japan Marrow Donor Program (JMDP), Tokyo, Japan; 19 Department of Hematology and Medical Oncology, University Hospital, Leipzig, Germany; 20 Lithuanian University of Health Science, Kaunas, Lithuania

**Keywords:** Stem cells, Health care

## Abstract

Hematopoietic cell transplantation (HCT) activity is increasing at an unprecedented pace with > 50,000 allogeneic transplants occurring annually worldwide. Establishing a functional HCT donor registry can be very challenging with respect to ethnicities, financial, technical, and geopolitical issues. Extensive planning steps are essential to overcome the expected challenges while establishing the registry, and to maintain its functionality. A few strategies can help move past those challenges and push the development of such registries forward. Authorities involved in HCT donor registry establishment will have to balance the advantages and costs of such a project and accommodate the emerging alternatives such as cord blood or related haploidentical transplants. Miscalculations and incomplete understanding of the various aspects of the process can have tremendous impact on the optimization of a HCT donor registry especially in developing countries. Herein we present some challenges in establishing such a registry and present potential solutions.

## Introduction

Hematopoietic cell transplantation (HCT) is a well-established curative therapy for certain types of cancers, blood disorders, genetic diseases, immunodeficiencies, and autoimmune conditions. The success of allogeneic HCT (allo-HCT) depends in large part on the degree of human leukocyte antigen (HLA) matching between recipient and donor. The ideal donor for allo-HCT is a matched sibling donor (MSD) but the overall chance of finding a MSD in each family is only ~ 25–30% [1] in the western world. The first evidence of hematopoietic stem cell engraftment was reported in 1957 by E. Donnall Thomas [2], and the first successful syngeneic HCT was done in 1969 [3]. The first successful matched unrelated donor (MUD) transplant took place in 1973 [4] when a patient with inherited immunodeficiency received an allo-HCT from a donor identified as a match through a blood bank in Denmark. Using MUDs to perform allo-HCT has been life-saving opportunity for patients without a MSD. This was the driving force to develop unrelated donor registries around the globe. The UK started the world’s first bone marrow donor registry, established by the Anthony Nolan Trust (ANT) in 1974 [5]. The number of allo-HCTs continues to increase worldwide, however many patients in need are unable to receive a transplant because a donor is not available for different reasons. The National Marrow Donor Program (NMDP) in the United States has > 19 million donors (https://bethematchclinical.org) but the majority of them are Caucasian, and the chance of finding a donor for a patient from another ethnicity or race is low, partly because of the under representation of minorities and different ethnicities in these large registries, but also because of the differences in HLA haplotype frequencies. This highlights the importance of local registries because generally ethnic groups share unique common haplotypes [6–8] and thus the chance of finding a MUD becomes much higher using local registries. However, establishing a registry for a region or a country can be immensely challenging. This special article will summarize challenges faced by emerging registries at a country and regional level with special emphasis on alternate donor registries in developing countries.

## Challenges

**Fig. 1:**
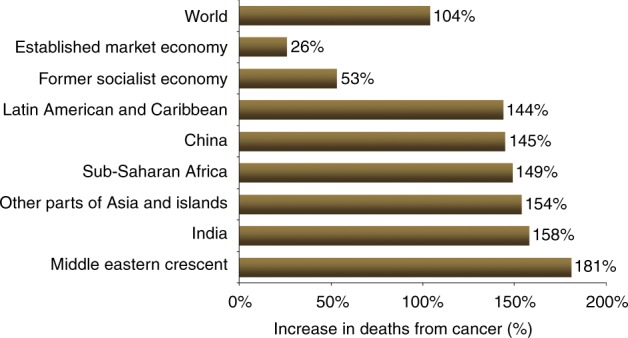
Expected cancer mortality rates in 2020

**Fig. 2:**
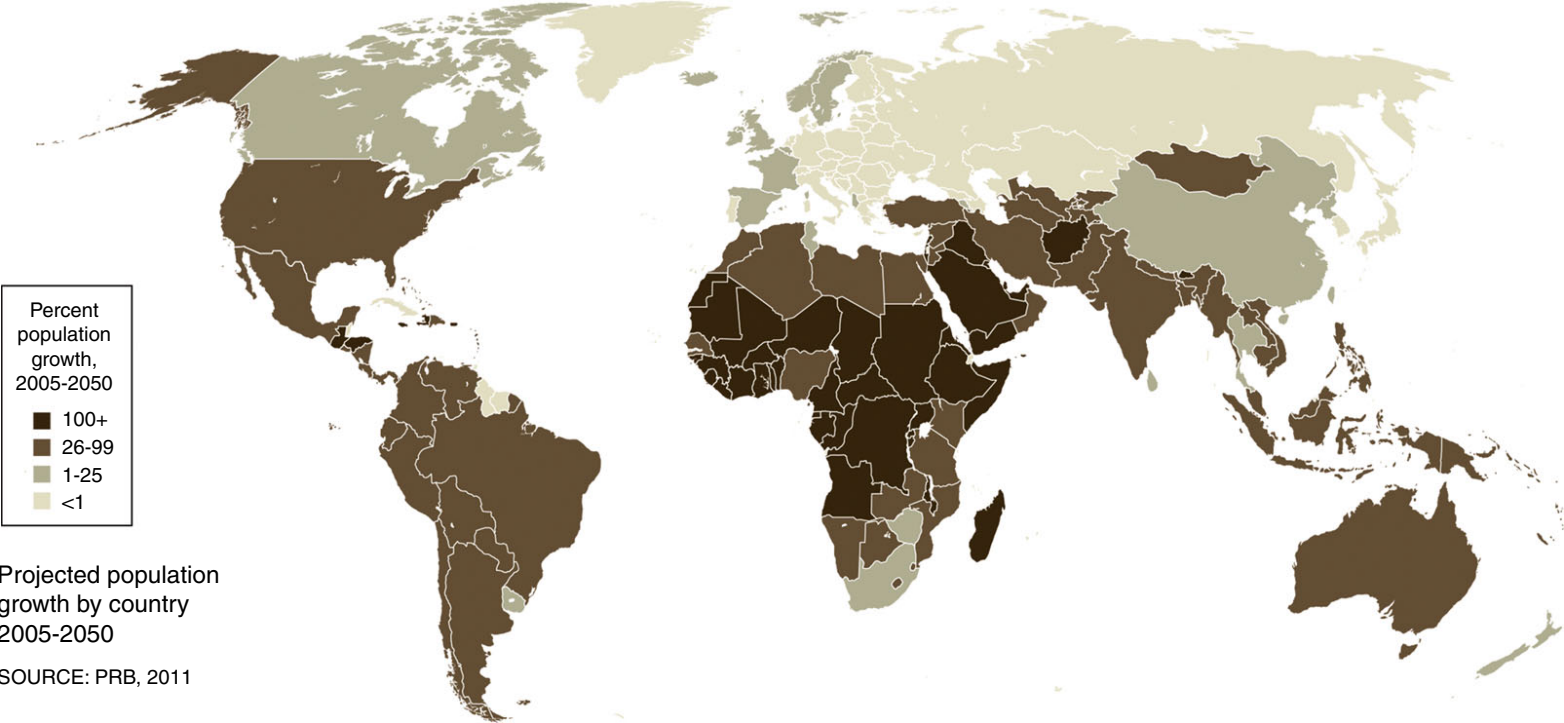
Projected population growth by country 2005–2050

**Fig. 3:**
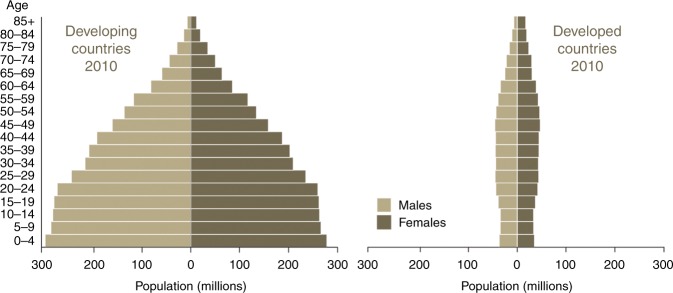
Age distribution in developing versus developed countries

**Fig. 4:**
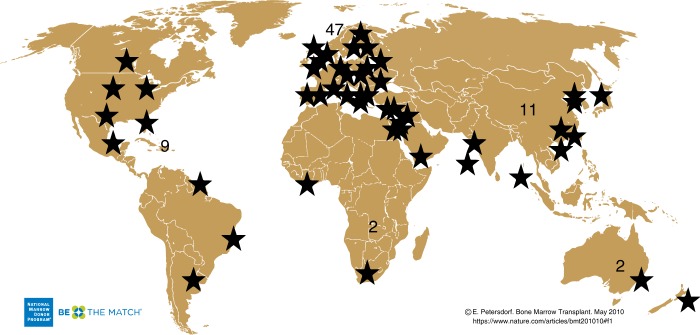
Location of unrelated donor registries worldwide

Cancer is one of the major causes of morbidity and mortality around the world; statistical models predict that in 2020 there will be 15 million new cancer cases, and that much of the cancer burden (mortality, incidence, and morbidity) will occur in the developing world [9]. Fig. 1 shows that cancer mortality will increase by 104% worldwide in 2020, and that the mortality rate would be fivefold higher in the developing world [10, 11]. This shift in disease burden is related to many factors including but not limited to poor access to advanced diagnostic and therapeutic modalities, research and epidemiologic data, cancer control, and prevention strategies [12, 13]. This, combined with the expected rapid and large population growth in developing countries [14] and changing population pyramids is alarming. Fig. 2 shows that the majority of the expected population growth will occur in developing countries. Fig. 3 shows the large proportion of younger people in these countries, which will accelerate the rapid growth in these countries and increase the demand on medical needs as compared with developed world countries. Fig. 4 shows the worldwide location of unrelated donor registries and outlines the limited number of registries outside North America, Europe, and East Asia. A comparison of Figs. 2–4 reflects the disproportionately limited number of donor registries in the developing world where most of the future growth and demand will be. These facts highlight the importance of adopting policies to close the gap and increase regional donor registries to facilitate transplants in “increasing-demand” countries. A first step toward correcting this problem is to understand the challenges faced when establishing a donor registry in these countries.

**Fig. 5:**
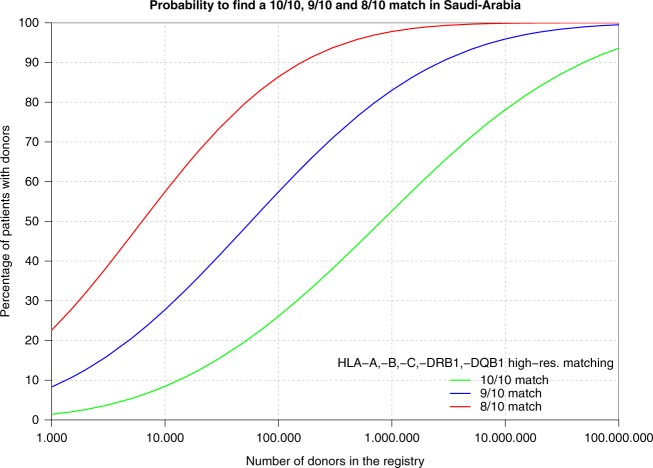
Probability to find a 10/10, 9/10, and 8/10 match in Saudi Arabia

1. Funding: maintaining a functional registry is expensive. Each step requires financial backup (donor recruitment, education events for the public, HLA typing (number of tested loci), ancillary tests (ABO, CMV, other infectious disease markers, etc.), laboratory maintenance, wages, donor collection facilities, software, etc.), plus the costs of maintaining contact with and communication with an increasing number of potential donors represent a significant financial burden for developing countries. This has led to the preferential and broad use of MUD HCT by high-income countries and rapid growth of related haploidentical transplantation as an alternative transplantation modality in lower income countries [15, 16]. A recent report from the Worldwide Network for Blood & Marrow Transplantation (WBMT) showed that MUD HCT is increasing worldwide with a preferential increase in high income countries (*P* = 0.02), with a clear association between transplant rates and Gross National Income per/capita (*P* < 0.01) [17]. Funding of a new regional or country registry is a major financial burden particularly in the early phases of establishing the registry and before the registry is functional in providing alternate donor grafts. Two pathways are traditionally followed, government funding (full or partial) and charity, either from private companies or patient support organizations.

2. Donor issues: emerging registries aim to improve donor availability for the country/region with its unique HLA phenotype spectrum. The needed registry size will depend on multiple factors including target population size, homogeneity of the population, and haplotype frequency distribution. India, for example, has a large, non-homogeneous population with > 300 distinct ethnic groups and 438 languages. In India, there are five organizations listing donors for international recipients: BMST India as an intermediary of DKMS Registry (21,695 donors), Be The Cure Registry-Jeevan Foundation (6449 donors), Datri Blood Stem Cells Registry (367,561 donors), GeneBandhu (7,991 donors), and the Marrow Donor Registry India (MDRI) (35,768 donors). (Source: https://statistics.wmda.info). However, the chance of finding a MUD at 10/10 low resolution level is only 19%, whereas the chances of finding a donor at high-resolution 10/10 level is even less [18]. Merging registries at the country level might offer greater efficiency. Mapping haplotype frequencies to regions in the country has allowed modeling future donor registry size [19]. Therefore, larger number of donors will be needed to be effective for patients in need of a transplant. Recruiting a large number of voluntary donors and minimizing donor attrition rates is difficult for multiple reasons, including lack of awareness, ethnic, religious, and other factors such as prevalence of infectious diseases in certain populations and BMI higher than 40, which is a relative exclusion criterion for donation. A US study showed that individuals from the white population are 30% more likely to donate compared with other racial groups and that some minorities have more religious and cultural objections to donate and report less trust in stem cell transplantation [20]. Awareness campaigns and counseling are good opportunities to help overcome fears, myths, and doubts, and thus maximize recruitment, donor commitment, and minimize attrition. Educating the public regarding various conditions that can be cured by transplantation may engage more volunteers. Recruiting regular platelet and blood donors is helpful as these donors are usually motivated to donate and already familiar with the apheresis procedure. Reaching out to key influencers and public figures in the society, collaborating with schools and organizations, using vaccination campaigns strategies, new educational tools and platforms, as well as social media may help to diffuse the mission of the alternate donor registry and expand registry size.

3. Haplotype frequency: HLA genes are very polymorphic with more than 18,000 alleles (http://hla.alleles.org/alleles/index.html) [21]. HLA loci have tight linkage and this characteristic has made HLA haplotype frequencies a useful tool to study population genetics, migration patterns, anthropological characteristics, as well as to determine the optimal composition and needed size for an effective registry [22, 23]. The mixing and ethnic diversity of a population increases the frequency of novel haplotypes and thus complicates the search for a compatible alternate donor. For instance, compared with the rest of the world, more than a third of HLA types within the Indian population are novel and unique [24]. On the other hand, in Saudi Arabia, a more homogeneous country, the chance of finding a 10/10 MUD is projected to be ~ 50% with a registry size of one million donors (Fig. 5) [25]. Another homogenous country is Japan where 8/8 HLA-matched donors are found for 96% of the patients [26]. In summary, the more heterogeneous the ethnic make-up of a country, the larger the registry size needed to ensure donor availability and registry cost-effectiveness. Computer programs to help estimating haplotype frequency are available to facilitate registry size planning [27]. Furthermore, HLA haplotype frequencies have a pivotal role for search algorithms designed to identify matching unrelated donors and are essential to estimate the probability of matching in a registry of a given size [19].

4. HLA typing: HLA typing by PCR testing was introduced by Mullis and Faloona in the 1980s [28]. Several PCR techniques are currently available, including DNA amplification with sequence-specific primers (SSP) [29], single-strand conformation polymorphism (SSCP) [30], sequence-specific oligonucleotide probes (SSO) [31], sequence-based typing (SBT) [32], DNA chip technology [33], and next-generation sequencing (NGS) [34–36]. These techniques offer better accuracy and are more reliable than serologic methods, and definitely help to standardize HLA-typing methods [37]. However, rapid progress in NGS technology has led to revolutionary changes in genomics. HLA testing using NGS provides both high-throughput and high-resolution capabilities as compared with PCR-based techniques. To date, several high-throughput HLA-typing methods using NGS have been developed [34–36]. It will be more practical for a new established registry to adopt NGS-based typing, and perhaps more cost effective to outsource HLA typing to reference laboratories to secure better pricing and accurate standardized testing. Several commercial laboratories are available and offer competitive prices. The method of sample collection for HLA typing (blood, finger stick, buccal swab, etc.) can have an impact on the amount of DNA recovered for testing and as such affect the failure rate of the test so that backup sampling is recommended.

5. Software: information technology (IT) is necessary to run donor searches, to analyze HLA-haplotype frequencies and to predict the chances of finding a donor for each patient, who usually desperately needs a transplant in the shortest possible timeframe. Historically, large international registries developed their own software with their internal IT staff and/or external software companies and some may have used blood bank software as their starting point. These in-house built programs are typically complex and tightly tailored to the local needs. They are not available commercially and thus not available for emerging registries. Building an in-house program needs a lot of effort, expertize, time, and expenses. This is particularly challenging for small and medium-sized emerging registries. Few commercial software packages for donor registries are now available in the market. Partnering with an established registry that has already developed an IT system is another way to acquire a software. Each of these models (home grown, commercial, partnership) has advantages and disadvantages (Table 1). The planning and preparation phase is the most critical phase. Ample time and a comprehensive team should be allocated to define the required functionality and ensure the security of the software. Once the scope of the system is decided (basic vs comprehensive), follow-up steps would include building a project team, studying the financial and quality requirements and then contracting with the suppliers. Table 2 summarizes the key steps for a successful planning phase. The ideal software should cover all key business processes of the registry daily work, including donor and patient data management, export to Search & Match Service of WMDA (previously known as BMDW), international donor search processes, management of requests, finances, transplant records and donor/patient follow-up. Table 3 outlines important elements for ideal registry software.

**Table 1:** Advantages and disadvantages of the currently available ways to acquire registry software^a^

Option	Advantages	Disadvantages	Example
Partnership: work with a ‘partner’ registry which has developed an IT system designed for registries.	Inexpensive. Partner has previously encountered potential difficulties.	The system may not support the business processes in your registry. Partner registry may always prefer their own interests and their own requests for change. IT support may be an issue.	The New Zealand Bone Marrow Donor Registry, Singapore Bone Marrow Donor Program and Thai Stem Cell Donor Registry use the Australian Bone Marrow Donor Registry software system.
Home grown: develop new software with the registry’s ‘in-house’ IT team.	Independence. “Made-to-measure” solution.	Costly and time-consuming. High level of expertize is needed. Often exceeds project plan and budget.	NMDP/Be The Match, ZKRD, France Greffe de Moelle (FGM), Italian Bone Marrow Donor Registry (IBMDR), Australian BMDR and others have their own IT team who develop their own software solutions.
Commercial system.	Clear customer–vendor relationship (deadlines, guarantees, budget limit). Experience of other users of the system. The supplier guarantees implementation of changes on schedules set by the community.	Limited number of vendors exists. The prices charged may be high if the registry has limited funding.	Registries in Belgium, Finland, Sweden, South Africa and the UK (BBMR) use a commercial system.

^a^Adopted from WMDA handbook with permission

**Table 2:** Initial planning phase for software acquisition^a^

The scope of the software (basic or comprehensive functionality)	–Basic: the WMDA Standards section 5.0 defines the minimum acceptable functionality of software. At its simplest the system must be able to register donors and patients, search a database of HLA data for volunteer donors and/or cord blood units, and provide search reports for transplant centers. –Comprehensive: manages all the registry’s functions with workflow support, modules for online communication and finance, interfaces to enable connection with other systems, a document management system, customer relationship management (CRM) function, automated production of letters, reports, and documents and the ability to monitor key performance indicators.
Project team	–The end users define the registry needs and as such are the cornerstone of the team; other key members of the team include: project manager, IT experts, administrators, quality management specialists, HLA experts, transplant physicians, donor recruitment staff, and finance department.
Contracting for suppliers	–Competence, reliability, and the role of the supplier are essential (install, configure, integrate, validate, maintain, modify, auditing, etc.). –Contractual obligations should clearly state conditions placed on both parties.
Financial aspects	–In addition to the initial cost recurring system costs include licensing fees, maintenance costs, and software updates.
Quality requirements	–There are a number of rules and regulations that should be considered regarding quality requirements for IT systems.^b^

^a^Adopted from WMDA handbook with permission

^b^EU Guidelines to Good Manufacturing Practice Medicinal Products for Human and Veterinary Use

**Table 3:** Elements of the ideal registry software^a^

Donor database	Patient database	Work process	Quality and security requirements
−Donor identification: unique registry ID for the donor is the primary reference (other IDs are allowed). In 2017, WMDA implemented the GRID (Global Registration Identifier for Donors), which will facilitate standardization of donors identification globally. −HLA data: separate fields for serology and DNA typing results, typing laboratory, date of typing, primary typing data, NIMA, etc. −Demographics: name, title, gender, date of birth, ethnic group, insurance company etc. −Relationships: family or personal relations to other donors or patients, used for family reports of the patient. −Recruitment: donor center, date of recruitment, method (website, patient-draft, blood donor, indication of whether the donor is also a blood or platelet donor). −Donor status: active or no, and if not active is the withdrawal temporary or permanent? What’s the reason of withdrawal (age, medical, personal)? –Contact details: permanent, temporary, or work address, email, phone numbers, social media networks, language, preferred contact method, history of communication with the donor. –Medical questionnaire: weight, height, blood group, hemoglobin, number of pregnancies, number of blood transfusions, donor consent to different types of donations, medical history. –Infectious disease markers: CMV status, toxoplasmosis, EBV status, HIV status, HIV-p24 antigen, antibodies to HIV, hepatitis B and C status and antibodies, syphilis status with dates of tests, and laboratories that performed tests. –Products: information about the stored donor samples. –Cord blood unit data: volume of cord blood unit, total nucleated cells, CD34 + cells, mononucleated cells, white blood cells, processing methods, fractions, and maternal tests. −Collection: date and place of collection, date, and place of transplant, patient ID, source of stem cells (bone marrow, PBSC, DLI, cord blood, other). −Audit: who has created or modified the donor record and when, searchable history of changes to the donor record.	−Patient identification: unique registry ID for the patient is the primary reference (other IDs are allowed). −HLA data: separate fields for serology and DNA typing results, typing laboratory, date of typing, primary typing data. Separate fields for historical HLA results. −Demographics: name, title, gender, date of birth, ethnic group, insurance company etc. −Relationships: family or personal relations to donors, used for family reports of the patient. −Patient status: donor search status, transplantation status, closure of the case (date, reason). −Medical information: diagnosis, disease phase, weight, blood group, CMV status. −Transplants: date and place of collection, date and place of transplantation, donor ID, source of stem cells. −Quality control: the system should control quality of data according to registry policies. There should be no expired reservations of donors , no over-age donors that are marked as ‘available for transplant purposes’ on the searches, no donors missing critical data (e.g., date of birth, gender) and HLA data should always be valid according to the latest HLA nomenclature. −Regular update of reference tables: for HLA nomenclature and multiple-allele-codes. −Reports: customizable reports of donor and patient details, ability to export to PDF files, ability to send letters and emails to donors through user-defined templates. −Transplant records, donor and patient follow-up records with automated reminders of incomplete or missing records. −Audit: who has created or modified the patient record and when, searchable history of changes of the patient record.	−Donor searches: a validated donor search algorithm is the key and most difficult element of the registry software. It has to be compliant with the WMDA guidelines. −Output report: a search report displaying the donors and cord blood units in HLA matching order, as a minimum. Only active donors should appear in search reports. −Management of requests (electronic, fax, or papers): typing requests, verification typing sample requests, infectious disease marker testing requests, donor reservation requests and work-up requests. The software must be able to display the status of the request. The system should support the workflow. management of requests for different scenarios. −WMDA annual report: Many registries do not systematically collect data for the WMDA annual report. There is a huge advantage to building in this functionality to generate data automatically at the start of the IT project. This will also help to perform annual statistics and research. −Financial module: creation of invoices according to registry policy and behavior of users. Export of invoices to financial system can be integrated into the request management workflow. Integration with external financial software systems. −Management of code lists (insurance companies, diagnosis, postal codes, etc.). −Document management system: possibility to store and maintain different kinds of electronic documents, linked to donor, patient, search, and other types of records. −International interfaces: the registry should be well integrated to the international community (BMDW, EMDIS^b^, EMDIS cord, NMDP, Netcord), mainly to assist with efficient donor searches. −National interfaces: the registry should be well connected with donor centers, collection centers, transplant centers, HLA laboratory. −Donors interface: it helps a registry to keep in contact with donors, Sponsors, social media networks. −HLA: regular import of the current HLA nomenclature. HLA data should always be valid according to the latest HLA nomenclature. −The software should support national names and characters. − Flexibility and availability of the company to update the software is crucial. −Generate letters and emails.	−The security of the overall data system should be compliant with WMDA Standards. −Authentication and authorization policies: to configure different user roles and limit the functionalities available to certain roles. The system should have different levels of access for different users. −Archiving of the data: including log-files, for 30 years or according to national legislation. −Traceability of changes: clear log-file of data saving, handling, changes, and removal of data, including what amendments were made, by whom, when the amendment took place and why. (Traceability is defined in the WMDA standards 1 as ‘to follow all the steps of a process from beginning to end). −Confidentiality: the access and transmission of data must be organized in a way that accidental or unauthorized access, destruction or modification of data is prevented and confidentiality is guaranteed. It is advisable at the outset of creating a new IT system to document how records will be protected from accidental or unauthorized access, destruction and modification. −Back-ups and disaster recovery planning: The registry must have a written policy on how its system is backed up. Back-ups should be performed regularly on a fixed schedule and stored off-site at some distance from the registry’s location.^c^ The registry should have contracts with hardware suppliers to provide replacement hardware within an agreed timeframe. −Electricity power backup system and internet backup system should be documented. −Documentation: all registries must have documents to describe key computer systems and network infrastructures. −Data quality control: the system automatically checks donor database every night and warns users about discrepancies that breaks registry policy. −All incidents should be reported and assessed. The root cause of a critical incident should be identified and should form the basis of Corrective And Preventive Actions (CAPA). −Under GMP regulations, the registry system should be periodically evaluated to confirm the IT system remains in a valid state and is GMP-compliant.

^a^Adopted from WMDA handbook with permission

^b^EMDIS (European Marrow Donor Information System) is an open computer network for the exchange of data between different stem cell donor registries

^c^WMDA Crisis Response, Business Continuity, and Disaster Recovery Guidelines, issued by WMDA Working Group Quality and Regulation

**Fig. 6:**
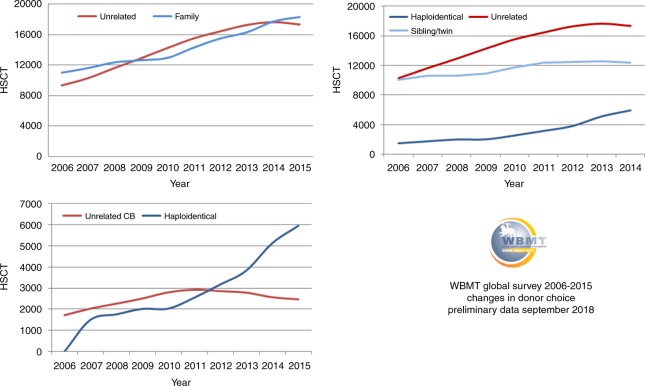
Changes in donor choice 2006–2015

6. Emerging alternatives: outcomes after HCT from related haploidentical donors (siblings, children, and parents) have improved significantly over the past few years, with results approaching those of MUD and MSD transplants. The use of haploidentical HCT will extend the availability of allo-HCT to virtually all patients in need, as almost all patients will have an available haploidentical related donor. This advantage, in addition to the low cost and immediate availability, is particularly important for countries with no donor registries and where cost is prohibitive for acquiring unrelated donor cells from international registries or for establishing a national registry. All of these facts suggest that in countries with a relatively small population and some ethnic and genetic diversity, establishing an alternate donor registry and making matched grafts available for a very limited number of patients per year may not be cost effective unless the country’s registry becomes part of an effective regional or international network for unrelated donors. Improvements in haploidentical HCT outcomes in the past decade, in particular using post-transplant cyclophosphamide, is making this alternative HCT modality preferred, over other alternative donors. Haploidentical family donor transplants are more affordable and readily available and therefore broadly used in many centers worldwide (Fig. 6), with a greater impact providing donors for patients in countries with limited resources. The availability of haploidentical HCT as alternative transplant modality has slowed the momentum toward establishing new regional registries or new cord blood banks.

7. Cord blood banks: Umbilical cord blood (UCB) banks provide an attractive, off the shelf solution for patients in need of an HCT. Recent studies showed comparable rates of acute graft-versus-host disease (GVHD) and significantly lower rates of chronic GVHD with cord blood transplant as compared to conventional matched donor transplant [38–41]. Despite this, the emergence of haploidentical HCT led to steadily decline in the use of UCB since 2010 worldwide (Fig. 6) except for Japan (http://www.jdchct.or.jp/en/data/slide/2017/). The disadvantages of using UCB are mainly the low yield of stem cells, resulting in delayed recovery, and the cost of graft acquisition from the international network of cord blood banks.

8. Utilization of artificial intelligence: artificial intelligence (AI) particularly with machine learning (ML) technology is revolutionizing the field of HCT in the selection of donors, and predicting outcomes after allo-HCT and even in GVHD [42–44]. The use of AI for donor–recipient pair matching in unrelated donor registries has been evaluated by multiple investigators and some have proven to increase accuracy of predictions for matching as well as for outcomes [45–47]. Although ML is perceived by some providers as a costly product, in fact, abundant data exist on predictions on how ML will reduce both costs and staffing along with improving clinical outcomes. As developing the ML modeling (especially via neural networks) for databases is done in collaboration with AI software engineers, a large number of startup companies exist that can help in developing algorithmic approaches for both active data management and for donor selection. Some of the developing countries adopting AI technology in healthcare include Morocco, Cameroon and South Africa (by integrating SOPHiA artificial intelligence for clinical genomics), and Rwanda (world’s first national drone delivery network for delivery of blood to remote areas for transfusion). Thus, early adoption of AI in establishing databases is both feasible and recommended.

## Conclusion

Although there has been a significant increase in the use of haploidentical related donors and unrelated cord blood (especially in Japan) as the stem cell source, the role of HCT from unrelated donors is well established. Establishing a functional and sustainable donor registry in a constantly evolving world is challenging, especially in the setting of limited resources and competition from emerging alternatives. Some existing registries are neither efficient nor able to provide suitable donors. Different causes of inefficiency exist, and all converge on the fact that resources may not be appropriately directed leading to poor performance and low yield of suitable donors. Analyzing the various steps in the process of recruitment, testing, retention until requested for donation may help in preventing donor attrition and thus increase the chance of building a successful registry. Educating the public may result in committed donors with low attrition rate, having a smart and easy-to-update software especially utilizing artificial intelligence algorithms, will ensure new HLA types are recorded and new search algorithms are built. Development of registries with widespread certified donation facilities will ease up the donation process. All of these are essential components for a functional registry. However, what remains both crucial and critical is the decision to build a registry or not, and that decision depends on the needs of the target population and the accessibility to alternative therapeutic options.
